# Fertility Does not Quarantine: Coronavirus Disease 2019 Pandemic Impacts on in Vitro Fertilization Clinical Pregnancy Rates

**DOI:** 10.1055/s-0043-1768459

**Published:** 2023-04-27

**Authors:** Fernanda de Almeida Vieira, Ricardo Pasquini Neto, Mariana Cristina Gomes Morila, Jean Borges Curimbaba, Daniela Sasso Pasquini, Paulo Cesar Zimmermann Felchner, Gustavo Wandresen, Jan Pawel Andrade Pachnicki

**Affiliations:** 1 Faculty of Medicine, Pontifical Catholic University of Paraná, Curitiba, PR, Brazil; 2 Medical Service, Brazilian Army, Curitiba, PR, Brazil; 3 Faculty of Medicine, Positivo University, Curitiba, PR, Brazil; 4 Department of Human Reproduction, Embryo Human Reproduction Center, Curitiba, PR, Brazil; 5 Department of Tocogynecology, Faculty of Medicine, Federal University of Paraná, Curitiba, PR, Brazil; 6 Department of Gynecology, Faculty of Medicine, Mackenzie Evangelical College of Paraná, Curitiba, PR, Brazil

**Keywords:** assisted reproductive techniques, fertilization in vitro, pregnancy, COVID-19, SARS-CoV-2, tecnologias de reprodução assistida, fertilização in vitro, gestação COVID-19, SARS-CoV-2

## Abstract

**Objective** To understand the impact of the coronavirus disease 2019 pandemic on in vitro fertilization (IVF) clinical pregnancy rates and analyze factors that may have influenced their outcome.

**Methods** This was a retrospective observational study conducted at a tertiary-care Brazilian fertility center. All fresh IVF and embryo warming cycles performed from March 11 to December 31, 2018–2021 were analyzed, and their data were used to calculate fertilization, embryo cleavage, cycle cancellation, embryo transfer (ET), and clinical pregnancy rates. Statistical tests were used to evaluate the alterations found. Logistic regression models were used to explore the association of the categorical variables with the observed clinical pregnancy rates. Data from 2018 and 2019 (prepandemic) and 2020 and 2021 (pandemic) were grouped.

**Results** A total of 756 cycles were analyzed ( *n* = 360 prepandemic and *n* = 396 pandemic). The age group of the patients, fertilization rates, and cleavage rates did not have significant differences ( *p* > 0.05). There was a reduction in the percentage of fresh IVF and an increase in embryo warming cycles ( *p* = 0.005) during the pandemic. There was also an increase in fresh cycle cancellations ( *p* < 0.001) and a reduction in ET rates ( *p* < 0.001). The pandemic had a negative impact on clinical pregnancy rates ( *p* < 0.001) especially due to the increase in fresh cycle cancellations ( *p* < 0.001).

**Conclusion** Embryo warming cycles with subsequent frozen-thawed ET were presented as a viable alternative to continue assisted reproductive treatments against pandemic restrictions on fresh cycles, ensuring clinical pregnancy, albeit at a lower rate than that of the prepandemic period.

## Resumo

**Objetivo** Compreender os impactos da pandemia de COVID-19 nas taxas de gravidez clínica em fertilização in vitro (FIV) e analisar fatores que possam ter influenciado seu resultado.

**Métodos** Foi realizado um estudo observacional retrospectivo em um centro brasileiro de reprodução assistida. Todos os ciclos de FIV com embriões frescos e descongelados realizados entre 11 de março e 31 de dezembro, 2018-2021 foram analisados, e seus dados utilizados para cálculo das taxas de fertilização, clivagem embrionária, cancelamento de ciclos, transferência de embriões (TE) e gravidez clínica. Testes estatísticos avaliaram significância das alterações encontradas e modelos de regressão logística exploraram associação das variáveis categóricas estudadas com as taxas de gravidez clínica observadas. Os dados de 2018 e 2019 (pré-pandemia) e 2020 e 2021 (pandemia) foram agrupados.

**Resultados** Foram analisados um total de 756 ciclos ( *n* = 360 na pré-pandemia e *n* = 396 na pandemia). A faixa etária das pacientes e as taxas de fertilização e de clivagem não tiveram alterações significativas ( *p* > 0,05). Na pandemia, houve redução da porcentagem de ciclos de FIV com embriões frescos e aumento dos com descongelamento ( *p* = 0,005). Também foi notado aumento das taxas de cancelamentos de ciclos com embriões frescos ( *p* < 0,001) e redução do número de TEs ( *p* < 0,001). A pandemia exerceu impacto negativo na taxa de gravidez clínica ( *p* < 0,001), especialmente devido ao aumento de cancelamentos dos ciclos a fresco ( *p* < 0,001).

**Conclusão** Frente às limitações pandêmicas impostas aos ciclos com embriões frescos, os ciclos de descongelamento de embriões se apresentaram como alternativa viável à continuidade dos ciclos de FIV, garantindo gravidez clínica ainda que em taxas inferiores às do período pré-pandêmico.

## Introduction

Since the first report of severe acute respiratory syndrome coronavirus 2 (SARS-CoV-2) in the city of Wuhan (China) in December 2019, the disease has spread rapidly and was characterized as a pandemic by the World Health Organization on March 11, 2020. 1 2 There was a need for emergency measures to contain transmission, mitigate the risk of community contamination and avoid the collapse of health systems. 3 4

Health authorities have advised the suspension of elective procedures, including the performance of new in vitro fertilization (IVF) treatment cycles. 5 6 The American Society of Reproductive Medicine and the European Society for Human Reproduction and Embryology published guidelines recommending a cryopreservation approach for patients who needed more urgent treatment. 7 8

In vitro fertilization is an assisted reproduction technology that consists of ovarian stimulation and egg capture, forming embryos that will be cultured, selected, and transferred into the uterus of infertile patients. In vitro fertilization can be performed by fresh cycles with immediate embryo transfer (ET) or through embryo warming cycles initially using the freeze-all embryo approach followed by the frozen-thawed ET (FET) at an opportune time. 9 For better effectiveness, the treatment must be performed at the right time, depending on the individual clinical condition of each patient ; delay in its initiation significantly reduces the probability of pregnancy, as well as causes psychological suffering. 3 5 10

Estimates indicate that > 1.5 million IVF cycles are performed each year worldwide, resulting in ∼ 400,000 live births. 11 Of all babies born each year in the UK and in the US, ∼ 3 and 2%, respectively, are conceived through assisted reproduction technologies. 12 13 14 Studies also indicate that the number of children who could be born by artificial methods, if there were no restrictions, could be as significant as the total number of deaths attributed to coronavirus disease 2019 (COVID-19). 14

The present study aimed to understand the impact of the COVID-19 pandemic on IVF clinical pregnancy rates and analyze factors that may have influenced their outcome.

## Methods

This was a retrospective observational analysis of medical records from a single tertiary-care fertility center located in Curitiba, state of Paraná, Brazil. Data from all patients who underwent IVF procedures (fresh and embryo warming cycles) from March 11 to December 31, 2018–2021, were included. No exclusion criteria were applied.

The following information was collected: mean age, number of cycles, IVF cycle pattern (fresh or embryo warming) performed, amount of fresh ETs and FETs, and clinical pregnancies observed. The number of cancellations was also analyzed when the cycles were interrupted before ET. Information regarding the number of recovered oocytes per cycle, the number of fertilized oocytes (with formation of two pronuclei), and the number of cleaved embryos were also collected. Data from medical records were extracted using GoldenSkill software.

Based on the data collected, clinical pregnancy, ET, and cycle cancellation rates were calculated. Clinical pregnancy rate was defined as the number of pregnancies diagnosed by ultrasonographic visualization of one or more gestational sacs, yolk sacs, and embryos over the number of cycles initiated. The ET rate consisted of the number of fertilized ET divided by the number of initiated cycles. The cycle cancellation rate corresponded to the number of interrupted cycles before ET over the number of cycles. 15

Fertilization and embryo cleavage rates were calculated from laboratory data. The fertilization rate consisted of the number of fertilized oocytes (with the formation of two pronuclei) relative to the number of oocytes retrieved. The cleavage rate was described as the number of embryos cleaved in relation to the number of oocytes with two pronuclei formed. According to the Brazilian national embryo production system protocol, these indicators have been used as efficiency parameters in assisted reproduction, reflecting the quality of oocyte/embryonic manipulation, laboratory inputs, and IVF laboratory environment. 16

The study followed the ethical principles of the Declaration of Helsinki and was approved by the research ethics committee of the local institution (CAAE:45576221.6.0000.0020). Since the study was retrospective and there was no direct contact with patients, informed consent was waived, in accordance with resolution 466/2012 of the Brazilian National Research Ethics Commission.

The population of the present study corresponded to the estimated 175,606 IVF cycles performed in Brazil between 2018 and 2021. This estimate was calculated from the 12 ^th^ and 13 ^th^ Brazilian national embryo production system protocols, which revealed the performance of 43,098 and 44,705 IVF cycles in Brazil in 2018 and 2019, respectively. 16 As the protocol was not updated during the pandemic period, the study assumed that the number of cycles performed during the pandemic remained similar to the prepandemic period. The calculated sample size was 384 cycles.

The data obtained were organized in a Microsoft Excel (Microsoft Corporation, Redmond, WA, USA) spreadsheet and described as means and standard deviations (SDs) for quantitative variables and as frequencies and percentages for qualitative/categorical variables.

The authors grouped the data from 2018 and 2019 as the prepandemic group, and 2020 and 2021 as the pandemic group.

Initially, the Kolmogorov-Smirnov test was performed to verify the normal (Gaussian) distribution of the sample. Based on the results obtained, the Student t-test for independent samples was applied for parametric continuous variables ( *p* > 0.05) or the Mann-Whitney test for nonparametric continuous variables ( *p* < 0.05). The Pearson chi-squared test and the Fisher exact test were used for categorical variables.

Logistic regression models were adjusted for univariate and multivariate analyses of associations between independent categorical variables analyzed with a *p* < 0.2 and clinical pregnancy rates (dependent variable). A Wald test was used to assess the significance of each variable. The odds ratio (OR) was used as the estimated association measure.

The statistical analysis assumed a confidence level of 95% and a standard error of 5%. Differences were considered statistically significant at *p* < 0.05. IBM SPSS Statistics for Windows, Version 27.0 (IBM Corp., Armonk, NY, USA) was used for statistical calculations and inferential analyses.

## Results

**Fig. 1:**
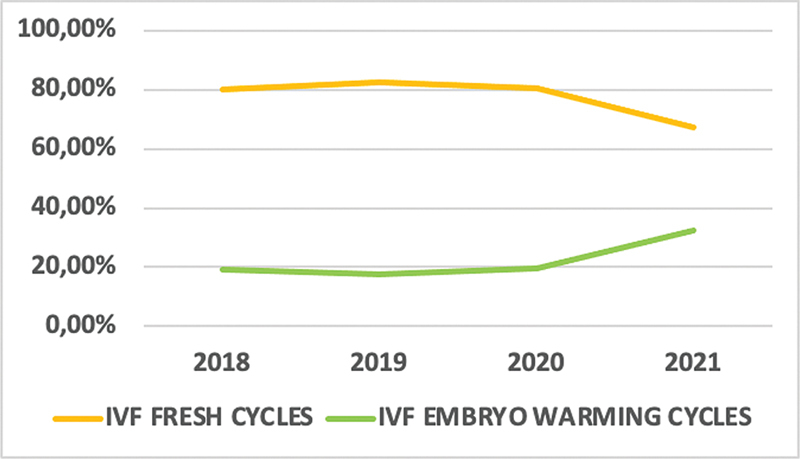
Behavior changes in IVF (fresh and embryo warming cycles) during the COVID-19 pandemic. IVF: in vitro fertilization; COVID-19: coronavirus disease 2019

A total of 756 IVF cycles were analyzed, including 360 during the prepandemic period and 396 during the pandemic. Of these, 590 (78.04%) were fresh cycles (297 [82.5%] in the prepandemic group, and 293 [73.99%] in the pandemic group) and 166 (21.96%) were embryo warming cycles (63 [17.5%] in the prepandemic group, and 103 [26.01%] in the pandemic group) ( *p* = 0.005) ( Figure 1 ).

**Fig. 2:**
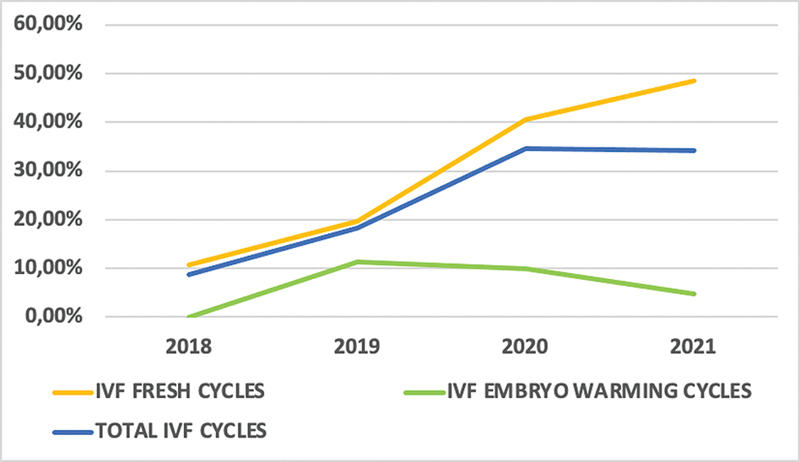
Behavior changes in IVF cancellation rates (fresh and embryo warming cycles) during the COVID-19 pandemic. IVF: in vitro fertilization; COVID-19: coronavirus disease 2019

Female age was 40 ± 0.8 years old prepandemic and 39 ± 0.7 years old during the pandemic ( *p =* 0.423). The percentage of women ≥ 35 years old was 87.83%, with 319 (88.61%) in the prepandemic period and 345 (87.12%) in the pandemic period ( *p =* 0.466). In the laboratory characteristics of the IVF fresh cycles, there was a recovery of 2,026 oocytes in the prepandemic period and 1,836 oocytes in the pandemic period ( *p =* 0.761); the fertilization rate was 75.7% in the prepandemic period and 76.2% in the pandemic period ( *p =* 0.744); the cleavage rate was 86.25% in the prepandemic period and 92.15% in the pandemic period ( *p =* 0.122). The embryo warming cycles laboratory variables were not available for analysis. During the analysis period, 184 (24.34%) IVF cycles were canceled (48 [13.33%] in the prepandemic group, and 136 [34.34%] in the pandemic group) ( *p* < 0.001). The cancellation rate of fresh cycles was 44 (14.81%) in the prepandemic period, which increased to 129 (44.03%) in the pandemic period ( *p* < 0.001). As for the embryo warming cycles, 4 (6.35%) cycles were canceled in the prepandemic period and 7 (6.8%) in the pandemic period ( *p =* 0.593) ( Figure 2 ).

**Fig. 3:**
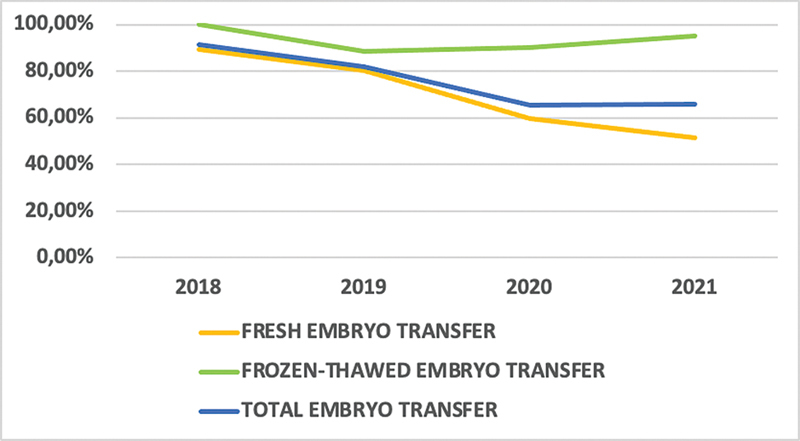
Behavior changes in IVF fresh and frozen-thawed embryo transfer rates during the COVID-19 pandemic . IVF: in vitro fertilization; COVID-19: coronavirus disease 2019

A total of 572 (75.66%) IVF cycles involved ET, 312 (86.66%) in the prepandemic group versus 260 (65.66%) in the pandemic group ( *p* < 0.001); regarding the fresh ET approach, there were 253 (85.18%) versus 164 (55.97%) ( *p* < 0.001); as for the FET approach, there were 59 (93.65%) versus 96 (93.2%) ( *p =* 0.482) ( Figure 3 ).

**Fig. 4:**
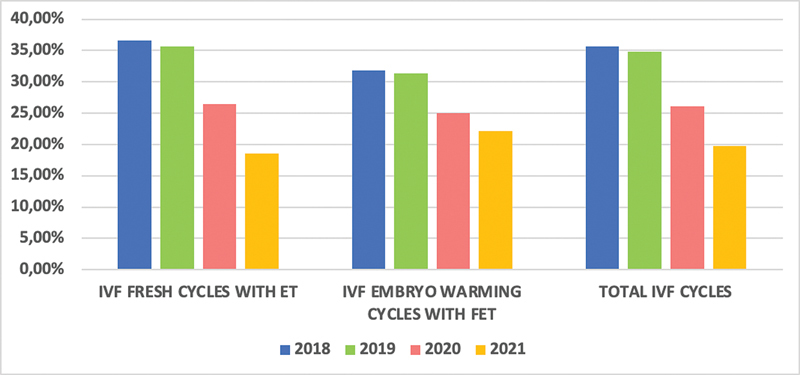
Behavior changes in IVF clinical pregnancy rates (fresh cycles with ET and embryo warming cycles with FET) during the COVID-19 pandemic. IVF: in vitro fertilization; ET: embryo transfer; FET: frozen-thawed embryo transfer; COVID-19: coronavirus disease 2019

Regarding the clinical pregnancy rates per cycle, 127 (35.28%) were in the prepandemic period versus 91 (22.98%) in the pandemic period ( *p* < 0.001); pregnancies in fresh cycles with ET were 107 (36.03%) versus 67 (22.87%) ( *p* < 0.001); pregnancies in embryo warming cycles with FET were 20 (31.75%) versus 24 (23.3%) ( *p =* 0.278) ( Figure 4 ).

Among the analyzed categorical variables, logistic regression identified that the cancellation of IVF fresh cycles was the only factor related to a significant reduction in clinical pregnancy rates during the pandemic (OR 0.052; *p* < 0.001). The other variables did not show any statistical significance.

## Discussion

At the beginning of the COVID-19 pandemic, studies focused on the SARS-CoV-2 respiratory effects and multisystemic inflammatory syndrome. 17 It was only during the second wave of the disease in Canada that Madjunkov et al. 18 synthesized the effects of COVID-19 on biological and assisted reproduction. This review pointed to the possibility of viral tropism to angiotensin-converting enzyme 2 receptors expressed in male and female reproductive organs. It also highlights the importance of future studies that measure in precise numbers the repercussions of the pandemic on assisted reproduction technologies, considering that the observed impacts have a potential negative effect on maternal-fetal-neonatal health. 18

In this context, the present study sought to fill the gap in the literature by quantifying the pandemic impacts on clinical pregnancy rates in women who underwent IVF fresh or embryo warming cycles. In Brazil, assisted reproductive technologies are not covered by the Brazilian unified public healthcare system (SUS, in the Portuguese acronym) or private health insurance plans. 19 Access by interested parties depends mainly on the availability of private financial resources, implying not only the cost of fertilization cycles but also the payment of complementary exams and the purchase of medications. 20

The data revealed considerable differences in IVF procedures during the pandemic. A significant increase in the total number of IVFs was associated with a significant change in the pattern of cycles. In line with health authorities imposing restrictions on suspending and postponing the start of new IVF fresh cycle treatments, there was a higher prevalence of embryo warming cycles and a reduction in fresh cycles. 5 6

In the context of restrictive measures, human reproduction societies recommend a cryopreservation approach, preferably in patients with malignancies, autoimmune, and hematological disorders, as well as in those who need gonadotoxic treatments. 7 8 However, women with an increased possibility of infertility (advanced age, low ovarian reserve, and/or a previous history of ovarian stimulation) were not included as priorities, which justifies the maintenance of the age pattern observed in patients who underwent IVF before and during the pandemic. 10 21 In a study by the Ethics Committee of the American Society for Reproductive Medicine, it was found that the delay in starting treatment in these patients has devastating outcomes similar to those selected by the authorities. 22 The epidemiological analysis of the literature indicates that the neglected group corresponds to ∼ 30 to 50% of patients seeking IVF. 14 23

The IVF fresh cycle laboratory variables (number of oocytes recovered, fertilization rate, and cleavage rate) did not show significant changes between the analyzed periods. Although the embryo warming cycle laboratory variables could not be analyzed, it is hypothesized that they have also remained unchanged due to the lack of updated reference protocols.

The present study also verified that the IVF clinical pregnancy rates at the analyzed center suffered a negative impact during the pandemic. Despite the significant reduction in uterine ET rates during this period, inferential analysis attributed the cancellation of fresh cycles to this result. The literature attributes the reason for cancellations to the health restrictions imposed as well as the economic recession inherent to the pandemic. 24 25

Cancellation of IVF cycles has also been identified in the literature as an important trigger of emotional stress in many patients seeking treatment for infertility. 26 27 Marom Haham 5 revealed that despite the risks of viral contamination and vertical transmission, most patients who had their IVF cycles suspended or postponed faced episodes of anxiety and frustration as they would still like to continue the treatment. The sharp decline in fertility and the reduction in IVF success in women > 35 years old may explain why patients in this age group feel more anxious to resume treatment during the pandemic.

In view of the outcomes of COVID-19 infection during pregnancy, no major concerns have been reported. Setti et al. 15 evaluated the outcomes of the first trimester of pregnancy in asymptomatic patients who were being treated with assisted reproductive technologies. The study did not demonstrate an increased risk of miscarriage, nor did it show other changes that were exacerbated during the pandemic. 15 Kotlyar et al. 28 described that the risk of vertical transmission in the 3 ^rd^ trimester of pregnancy occurs in a minority of cases without bringing greater complications to the fetus. The fear of viral contamination during pregnancy, with the possibility of harming the fetus, is not supported as a justification for the suspension of IVF cycles. 18

The reduction in pregnancy rates behaved in a peculiar way depending on the IVF cycle pattern and transfer method used. Although a systematic review and meta-analysis by Zaat et al. 9 showed an extremely small difference in pregnancy rates between the fresh ET and FET approaches, our study revealed that during the pandemic period, there was a significant reduction in pregnancy rates with the fresh ET approach, while those by FET remained unchanged. This represents the value of embryo warming cycles with FET as a viable alternative to fertility preservation in a scenario where fresh ET is limited. It is hypothesized that the reduction in pregnancy rates could have been much greater if the FETs had suffered more pronounced limitations. According to Madjunkov et al., 18 the embryo warming cycles with FET proved to be safe in avoiding viral contamination of the sample in cryopreservation laboratories due to the rigid air control systems and negative pressure chambers.

The limitations of the present study include its retrospective design, data collection from a single center, and the analysis conducted up to the moment of clinical pregnancy diagnosis. Due to unavailability of data, it was not possible to completely analyze the demographic characteristics of the patients, nor observe the evolution of pregnancy and neonatal outcomes. Thus, we encourage the performance of new multicenter studies that compare the impacts of the COVID-19 pandemic in different regions, as well as studies that evaluate the entire gestation period of patients, allowing for an understanding of the impacts of the pandemic on the quality of gestation and birth rate from IVF procedures.

## Conclusion

Based on the data analysis of a tertiary-care Brazilian fertility center, the present study identified that the pandemic had a negative impact on IVF clinical pregnancy rates, especially due to the significant increase in fresh cycle cancellation rates. The present study also highlights the value of embryo warming cycles with FET as a viable alternative to continue assisted reproductive treatments against pandemic restrictions. Although fresh cycles have been limited and many of them interrupted by health system overload that were focused on the exclusive care of COVID-19 patients, the FET approach ensured clinical pregnancy, albeit at a lower rate than the prepandemic period.
